# The Physical Diagnosis of Diseases of the Lungs

**Published:** 1843-01

**Authors:** 


### Art. XX.

*The Physical Diagnosis of Diseases of the Lungs.* By Walter Hayle Walshe, m.d., Professor of Pathological Anatomy in University College, London; Physician to the Hospital for Consumption, &c. &c. —*London*, 1843. 8vo, pp. 320.

Had this book reached us at an earlier period of our trimestral labours, we should have felt it our duty to give a detailed account of its contents, although they are of a kind which scarcely admit of further compression by analysis. Under present circumstances, we must content ourselves with a brief exposition of the nature and plan of the work, and a statement of the judgment we have formed of its merits after a careful perusal.

The following extracts from the preface and table of contents give a complete view of the author's plan.

"In the first of the three parts, into which the work is divided, the various methods of physical examination, and the phenomena detected by them in the states of health and of disease, are described. In the second part will be found a tabular view of the physical causes and ordinary seat of all morbid signs, in connexion with the names of the diseases in which they occur; and also a synopsis of the signs attending each affection of the lungs, pleura, and larynx. The third part forms a commentary upon the two preceding. By excluding from the descriptive portion any discussion upon debateable points, and in the tabular views by distinguishing with a different type the more striking phenomena from those less constantly useful and available in practice, I have, I trust, succeeded in adapting the book for beginners. The attention bestowed upon them in the Commentary will, however, show that I do not undervalue delicate signs: far from this, I know by experience that in many instances apparently trivial phenomena will justify a diagnosis that without their aid had been altogether unwarrantable. I have avoided, as far as possible, all disquisition upon the acoustic principles regulating the production and transmission of sounds; not on account of any deficiency of interest or importance on the subject, but because I was unwilling to increase the size of the book, or deprive it of the simple practical character I had aimed at giving it."

"Part I.—General description of the methods of physical diagnosis.

"Sect. I.—Inspection:—Results of inspection of the natural state; morbid states discovered by inspection.

"Sect. II.—Application of the hand:—Results of application of the hand in the natural state; morbid states discovered by application of the hand.

"Sect. III.—Mensuration:—General measurements; partial measurements.

"Sect. IV.—Percussion:—Results of percussion in the natural state; variations compatible with health; morbid states discovered by percussion.

"Sect. V.—Auscultation:—Natural respiratory murmurs; modified conditions of the respiratory murmurs; sounds superseding the respiratory murmurs; adventitious sounds; modified conditions of the respiratory murmurs in the trachea and larynx; resonance of the voice; natural vocal resonance; unnatural or morbid vocal resonance; resonance of the cough; phenomena common to the sounds of respiration of the voice and of the cough, sounds of the heart and vascular murmurs, as transmitted through the substance of the lungs.

"Sect. VI.—Succussion.

"Sect. VII.—Determination of the situation of surrounding parts and organs.

"Part II. § 1.—Table exhibiting the physical cause and ordinary seat of the different physical signs, together with the names of the diseases in which they are observed.

"Sect. I.—Signs discovered by inspection.

"Sect. II.—Signs discovered by application of the hand.

"Sect. III.—Signs discovered by mensuration.

"Sect. IV —Signs discovered by percussion.

"Sect. V.—Signs discovered by auscultation.

"Sect. VI.—Sign discovered by succussion.

"Sect. VII.—Displacements of surrounding parts and organs.

"§ 2.—Synopsis of the physical signs of the diseases of the lungs.

"Part III.—Commentary."

In the treatment of the subjects thus lucidly arranged, the author has shown great skill and judgment, and has displayed such a thorough practical familiarity with every one of them, as cannot fail to gratify every reader. True to his plan of furnishing a real practical guide to physical diagnosis, Dr. Walshe in the first two parts has not allowed himself to be seduced into any unnecessary details of matters of fact, or into any discussion on doubtful or disputed points; while in Part III, (*the Commentary*,) which occupies half the volume, he has treated, in the most perspicuous manner, and in the most condensed form, of almost everything—whether relating to theory or fact—which has engaged the attention of auscultators since the days of Laennec.

We regret that we are not able to give a series of continuous extracts as specimens of the different divisions of the book, to show the precise manner in which are they handled; and our readers must be content to take our word for what such specimens would demonstrate—that the treatise is one of extraordinary merit. Indeed we do not hesitate to say that there exists in no language any work on the physical diagnosis of diseases of the lungs, suited for students, so clear and precise, and at the same time so comprehensive and practical as this. It is one which no learner in auscultation can fail to possess, without losing advantages elsewhere unattainable; and it is one which very few even among the most experienced auscultators will consult without adding something to their previous stock of knowledge.

We trust the author intends soon to complete his plan by giving us the physical diagnosis of the diseases of the heart and great vessels; and we would recommend him to render the work still more perfect, by adding the physical diagnosis of the diseases of the abdomen.

